# Evaluation of a novel monoclonal antibody against tumor-associated MUC1 for diagnosis and prognosis of breast cancer

**DOI:** 10.7150/ijms.35452

**Published:** 2019-08-14

**Authors:** Natascha Stergiou, Johannes Nagel, Stefanie Pektor, Anne-Sophie Heimes, Jörg Jäkel, Walburgis Brenner, Marcus Schmidt, Matthias Miederer, Horst Kunz, Frank Roesch, Edgar Schmitt

**Affiliations:** 1 Institute for Immunology, University Medical Center;; 2 Institute for Nuclear chemistry, Johannes-Gutenberg University;; 3 Clinic and Polyclinic for Nuclear Medicine, University Medical Center;; 4 Department of Obstetrics and Women's Health, University Medical Center, Johannes Gutenberg-University, Germany,; 5 Department of Pathology, University Medical Center;; 6 Institute for Organic Chemistry, Johannes-Gutenberg University.

**Keywords:** MUC1, breast cancer diagnosis, mAb, ^89^Zr

## Abstract

There is still a great unmet medical need concerning diagnosis and treatment of breast cancer which could be addressed by utilizing specific molecular targets. Tumor-associated MUC1 is expressed on over 90 % of all breast cancer entities and differs strongly from its physiological form on epithelial cells, therefore presenting a unique target for breast cancer diagnosis and antibody-mediated immune therapy. Utilizing an anti-tumor vaccine based on a synthetically prepared glycopeptide, we generated a monoclonal antibody (mAb) GGSK-1/30, selectively recognizing human tumor-associated MUC1. This antibody targets exclusively tumor-associated MUC1 in the absence of any binding to MUC1 on healthy epithelial cells thus enabling the generation of breast tumor-specific radiolabeled immune therapeutic tools.

**Methods:** MAb GGSK-1/30 was used for immunohistochemical analysis of human breast cancer tissue. Its desferrioxamine (Df')-conjugate was synthesized and labelled with ^89^Zr. [^89^Zr]Zr-Df'-GGSK-1/30 was evaluated as a potential PET tracer. Binding and pharmacokinetic properties of [^89^Zr]Zr-Df'-GGSK-1/30 were analyzed *in vitro* using human and murine cell lines that express tumor-associated MUC1. Self-generated primary murine breast cancer cells expressing human tumor-associated MUC1 were transplanted subcutaneously in wild type and human MUC1-transgenic mice. The pharmacology of [^89^Zr]Zr-Df'-GGSK-1/30 was investigated using breast tumor-bearing mice *in vivo* by PET/MRT imaging as well as by *ex vivo* organ biodistribution analysis.

**Results:** The mAb GGSK-1/30 stained specifically human breast tumor tissue and can be possibly used to predict the severity of disease progression based on the expression of the tumor-associated MUC1. For *in vivo* imaging, the Df'-conjugated mAb was radiolabeled with a radiochemical yield of 60 %, a radiochemical purity of 95 % and an apparent specific activity of 6.1 GBq/µmol. After 7 d, stabilities of 84 % in human serum and of 93 % in saline were observed. *In vitro* cell studies showed strong binding to human tumor-associated MUC1 expressing breast cancer cells. The breast tumor-bearing mice showed an* in vivo* tumor uptake of >50 %ID/g and clearly visible specific enrichment of the radioconjugate *via* PET/MRT.

**Principal conclusions:** Tumor-associated MUC1 is a very important biomarker for breast cancer next to the traditional markers estrogen receptor (ER), progesterone receptor (PR) and HER/2-neu. The mAb GGSK-1/30 can be used for the diagnosis of over 90% of breast cancers, including triple negative breast cancer based on biopsy staining. Its radioimmunoconjugate represents a promising PET-tracer for breast cancer imaging selectively targeting breast cancer cells.

## Background

Breast cancer is the most common cancer among women worldwide and the leading cause of cancer death among women 1. One in eight women suffers from breast cancer in her life 2. Breast cancer is usually detected either during a check-up before symptoms develop or after a woman has discovered a cancerous lump. If cancer is suspected, a microscopic analysis of the breast tissue is required for diagnosis, determination of breast cancer status and type of breast cancer. The tissue for microscopic analysis can be obtained by fine needle biopsy or surgery (American Cancer Society, Breast cancer risk factors). Traditional molecular markers for the characterization of breast cancer are estrogen receptor (ER), progesterone hormone receptor (PR) and Her2/neu; the standard method for their global assessment remains immunohistochemistry 3. The results of biopsy analysis are important for prognostic and therapeutic considerations 3. Due to the heterogeneity of breast cancer, these traditional markers are often not sufficient either for a precise prognosis or a sufficient statement about an adjuvant or neoadjuvant therapy. It is therefore essential to look for additional prognostic and predictive breast cancer markers that will be complementary in predicting clinical response to the available therapeutic modalities. In addition, there is a need to develop additional markers for such tumors that do not express ER, PR and or HER-2/neu (triple-negative breast cancer [TNBC]) 4. The aim is to bring additional markers in the clinic that predict the risk of recurrence and are helpful in decision making regarding appropriate treatment [8]. In 8 to 10% of women diagnosed with breast cancer, locoregional recurrences occur, and 15 to 30% develop distant metastases 1. A very promising marker to support breast cancer diagnosis and prognosis is the tumor-associated MUC1 ((TA)MUC1) 5-9. It is expressed in over 90 % of all breast cancers 10 and even in 94 % of TNBCs 11. Due to its characteristic aberrant glycosylation as a result of reduced activity of glycosyltransferases and accelerated activity of sialyltransferases in the MUC1 biosynthesis in breast cancer cells 12, breast-(TA)MUC1 represents a tumor-specific marker and target for therapy 13. Based on the aberrant glycan pattern, we synthesized human (TA)MUC1 (hu(TA)MUC1) glycopeptides derived from the tandem repeat (VNTR) region of this glycoprotein that correspond to the aberrant glycosylation pattern of (TA)MUC1. One specific hu(TA)MUC1 glycopeptide, 22mer huMUC1 peptide sequence of the VNTR region glycosylated with ST_N_ on serine-17 located in the highly immune reactive GSTA motif, was conjugated to tetanus toxoid (TTox) forming an unique vaccine 5. Monoclonal antibodies (mAbs) were generated utilizing this vaccine. Among these, mAb GGSK-1/30 was identified that specifically recognized the hu(TA)MUC1-glycopeptide pattern on human breast cancer cells whereas fully glycosylated huMUC1 expressed by healthy breast epithelial cells was not recognized. We could also demonstrate that GGSK-1/30 showed stronger binding to these breast cancer cells than the commonly used and commercially available mAbs (SM3, HMFG1) 12. The aim of the current study was to evaluate the mAb GGSK-1/30 as a diagnostic and prognostic tool for breast cancer. Therefore, this mAb was evaluated by applying immune histochemical assays and molecular *in vivo* imaging using PET.

## Methods

### Monoclonal antibody GGSK-1/30

GGSK-1/30 was generated as described before after vaccination of BALB/c mice with a 22mer huMUC1 peptide sequence of the VNTR region coupled to TTox 5,12. GGSK-1/30 is of IgG1 isotype and was purified from hybridoma supernatant using Protein G and subsequently dialyzed versus PBS with the aid of a PD-10 desalting column (Sephadex G-25).

### Histological staining of human breast cancer specimens

A panel of 144 HR positive breast cancer tissue specimens of patients who were treated at the Department of Obstetrics and Gynecology of the Johannes Gutenberg University Mainz between the years 1987-2000 was examined for the expression of (TA)MUC1 by using GGSK-1/30 as a diagnostic tool. Patients' characteristics are given in **Table 1**. Immunohistochemical analyses were performed on 4 µm thick FFPE (Formalin-fixed paraffin embedded) sections according to standard procedures. In brief FFPE slides were subsequently deparaffinized using graded alcohol and xylene. Antigen retrieval reactions were performed in a steamer in citrate buffer of pH10 for 30 minutes. 3% H_2_O_2_ solution was applied to block endogenous peroxidase at room temperature for 5 minutes. The samples were stained with GGSK-1/30 (1 μg/ml), followed by a polymeric biotin-free visualization system reaction (EnVision™, DAKO Diagnostic Company, Hamburg, Germany). In a next step, the sections were incubated with 3,3-diaminobenzidine (DAB; EnVision™, DAKO Diagnostic Company, Hamburg, Germany) for 5 minutes and counterstained with Mayer's haematoxylin solution. Paraffin sections of healthy breast tissue and paraffin sections of HR positive breast cancer tumours were examined. All slides were analyzed using a Leica light microscope (Leica Microsystem Vertrieb Company, Wetzlar, Germany) by two of the authors (ASH, JJ). Additionally, the magnitude of expression of (TA)MUC1 was scored in correlation of cumulative MFS and RFS according to the scoring system of Sinn *et al.* 14. This work was approved by the Landesärztekammer Rheinland-Pfalz, 837.287.05 (4945). All patients gave written informed consent before participating in this study. Follow-up data of all patients until 2014 were available and included the time period until development of metastases.

### Evaluation of immunostaining

(TA)MUC1 expression was evaluated using an immunoreactivity score (IRS) as described by Sinn *et al.* 14 In brief, the percentage of positive tumor cells (0% = 0, 1%-10% = 1, 11%-50% = 2, 51%-80% = 3, 81%-100% = 4) and the staining intensity (negative = 0, weak = 1 moderate = 2, strong = 3) were multiplied, resulting in an immunoreactivity score (IRS) from 0 to 12. Cases with IRS 0-2 were considered as negative in terms of (TA)MUC1 expression due to possibly unspecific staining or material artifacts whereas cases with IRS 3-12 were considered as clearly visible and analyzable (TA)MUC1 expression. Kaplan-Meier Plots were performed to estimate survival rates. Significance levels were calculated using Log-Rank-Test. Statistical analysis was performed using the Graphpad Prism statistical software program, version 8.0.

### Animal breeding

The transgenic C57BL/6-TG(MUC1)79.24GEND/J 15 mice (huMUC1-tg, The Jackson laboratory) transgenically express the human MUC1 gene (huMUC1) and were housed and maintained in microisolator cages under specific pathogen-free conditions at the animal facility of Johannes Gutenberg-University following institutionally approved protocols (permission was obtained from the Landesuntersuchungsamt Koblenz, 23 177-07/G 08-1-019).

### Cell culture

To obtain stable tumor cell lines from the autochthonous tumors of female PyMTxhuMUC1 mice and female PyMT mice (age of 18 weeks), tumor tissues were extracted, digested by collagenase A (Roche, 2mg/ml) and RQ1 DNAse (Promega, 1:2000) and cultured in IMDM (PAN Biotech, Aidenbach, Germany) + 5 % FCS (Gibco^®^, Life Technologies, Carlsbad, USA) + 1 % glutamine (Roth, Karlsruhe, Deutschland) + 1 % sodium pyruvate (Serva, Heidelberg, Deutschland). Stable PyMTxhuMUC1 tumor cells, expressing hu(TA)MUC1 and PyMT tumor cells that do not express hu(TA)MUC1 could be harvested after 6 weeks. In the first two weeks the cells were washed every third day to remove the tissue residues. After that primary tumour cells were cultured for additional 4 weeks to obtain the outgrowing adherent tumour cells and were passaged every time at a confluency of 70%. Binding of GGSK-1/30 to both tumor cell lines (PyMTxhuMUC1 and PyMT) was analyzed via fluorescence-activated cell sorting (FACS) as follows: 2x10^5^ tumor cells were incubated with 1 µg/ml GGSK-1/30 for 20 min at 4 °C. The cells were washed two times with 100 µl of PBS and incubated for 20 minutes at 4 °C with a secondary antibody goat-α-mouse-IgG Alexa Fluor 488 (dilution 1:1000 in PBS) in combination with a fixable viability dye eFluor780 (dilution 1:1000 in PBS) to exclude false positive dead cells. Tumor cells were washed twice with 100 µl PBS followed by FACS analysis on a BD Biosciences FACSVerse machine.

### Conjugation of Df-Bz-NCS (Df') to GGSK-1/30 and radiolabeling with ^89^Zr

All used chemicals were commercially available at Acros Organics, CheMatech, Fluka, SigmaAldrich or VWR and were used without further purification. GGSK-1/30 was coupled with Df' following a known procedure 16. In short, a ten-fold molar excess of Df' (in 10 µl DMSO) was added to the GGSK-1/30 (2 mg/ml in 1 ml PBS set to pH 9.0 with 0.1 M Na_2_CO_3_) and incubated for 30 min at 37 °C. The chelator-GGSK-1/30 conjugate was purified by size exclusion chromatography (SEC) using a PD-10 column and 0.25 M sodium acetate buffer, pH 5.4 as eluent.

For purification of conjugated and radiolabeled antibody, PD-10 desalting columns (GE Healthcare Life Science) were applied for dialysis versus 0.9 % sodium chloride (Fresenius-Kabi) solution. For radiolabeling trace metal-free salts and water (18 MΩ cm^-1^) were used. For radiolabeling, no-carrier-added (n.c.a.) ^89^Zr (1 M oxalic acid) from PerkinElmer (Netherlands), trace metal-free salts and water (18 MΩ cm^-1^) were used.

### Determination of chelator-to-mAb ratio (CAR)

To determine the CAR, the conjugate was labeled according to aforementioned procedure 16 with a known nanomolar excess of zirconium oxalate solution (TraceCERT®, 1000 mg/ml) spiked with ^89^Zr. Different molar ratios between Zr and GGSK-1/30 mAb were used to determine the number of chelators per antibody.

### Preparation of [^89^Zr]Zr-Df'-GGSK-1/30 and analytical quality control of [^89^Zr]Zr-Df'-GGSK-1/30

Df'-GGSK-1/30 was labeled according to aforementioned method 17. In short, Df'-GGSK-1/30 was radiolabeled with ^89^Zr in HEPES buffer (0.5 M, pH 7) at room temperature in a volume of 2.5-3 ml under gentle stirring for 90 min. Radiochemical yield (RCY) was determined by radio thin layer chromatography (using Merck Silica F254 TLC plates with citrate buffer, (0.01 M, pH 4) analyzed with the radio detector GABI STAR (Raytest, **SI Figure 1**). The radiolabeled compound was purified by PD-10 column using a 0.9 % sodium chloride solution as eluent. HPLC monitoring was performed on a HPLC system from Merck (LaChrom; pump: Hitachi L7100; UV-detector: L7400) using a BioSep SEC-S 2000 column (Phenomenex®) with 0.05 M sodium phosphate (pH 7) as mobile phase (1 ml/min) (**SI Figure 2**).

### *In vitro* stability test of [^89^Zr]Zr-Df'-GGSK-1/30

*In vitro* stability studies of [^89^Zr]Zr-Df'-GGSK-1/30 were performed in human serum (Sigma-Aldrich®, from human male AB plasma) and sodium chloride (0.9 %) (n=3). The samples were incubated at 37 °C and aliquots of 2 µl were analyzed at various time points (1 d, 3 d, 7 d) via radio-TLC using citrate buffer (**SI Figure 3**).

### *In vitro* binding studies of [^89^Zr]Zr-Df'-GGSK-1/30

For *in vitro* binding studies different concentration of [^89^Zr]Zr-Df'-GGSK-1/30 (0.125-1 µg/ml) were incubated with 2x10^5^ tumor cells for 30 min at 37 °C. The supernatant was removed, the cell surface washed twice with PBS buffer. The washing solution was kept to detect the unbound antibody. Hence, the radioactivity of the cells and the washing solution was detected with a gamma counter (PerkinElmer Wizard2). The ratio cells/washing solution x 100 resulted in binding/%.

### Inoculation of tumor cells

For all *in vivo* kinetic experiments 10 weeks old female C57BL/6N mice (Janvier) were used. Either 1x10^6^ PyMTxhuMUC1 tumor cells or 2x10^5^ PyMT tumor cells were subcutaneously (s.c.) inoculated in the right flank. To determine the biodistribution of the mAb in mice expressing huMUC1 on every epithelial cell, mimicking the human background, 1x10^6^ PyMTxhuMUC1 tumor cells were inoculated in nine 10 weeks old huMUC1-transgenic mice. The tumor growth was observed every 3 days.

### Animal studies

21 d after inoculation (tumor size 40 mm^2^ on average), 50-80 µg (0.5-2.5 MBq ^89^Zr) of the radioconjugates were administered intraperitoneal (i.p.) in 250-300 µl PBS. Mice were anesthetized with isoflurane (2 vol%)/ oxygen gas mixture).

### *Ex vivo* biodistribution

All mice were sacrificed and dissected after 24 h, 48 h, 72 h and 10 d. Blood, tumor, normal tissue and gastrointestinal contents were weighted and the amount of radioactivity in each tissue was measured in a gamma-counter (PerkinElmer Wizard2). Radioactivity uptake was calculated as the percentage of the injected dose per gram of tissue (%ID/g(tissue)).

### *In vivo* small animal PET studies

Small animal PET studies were carried out 72 h after application of radioconjugates regarding the highest enrichment of the mAbs in the tumors at this time point. All scans were performed in head-first-prone position in a PET-MRI scanner (Mediso NanoScan, Mediso, Hungary). In some experiments MRI measurements (Material Map) were first performed for co-registration of the PET scan (3D Gradient Echo External Averaging (GRE-EXT), Multi Field of View (FOV); slice thickness: 0,6 mm; TE: 2 ms; TR: 15 ms; flip angle: 25 deg) followed by a static PET scan (collecting 20 million events). PET data were reconstructed with Teratomo 3D (4 iterations, 6 subsets, voxel size 0.4 mm), co-registered to the MR and analyzed with PMOD software (version 3.6, PMOD Technologies LLC).

## Results

**Figure 1:**
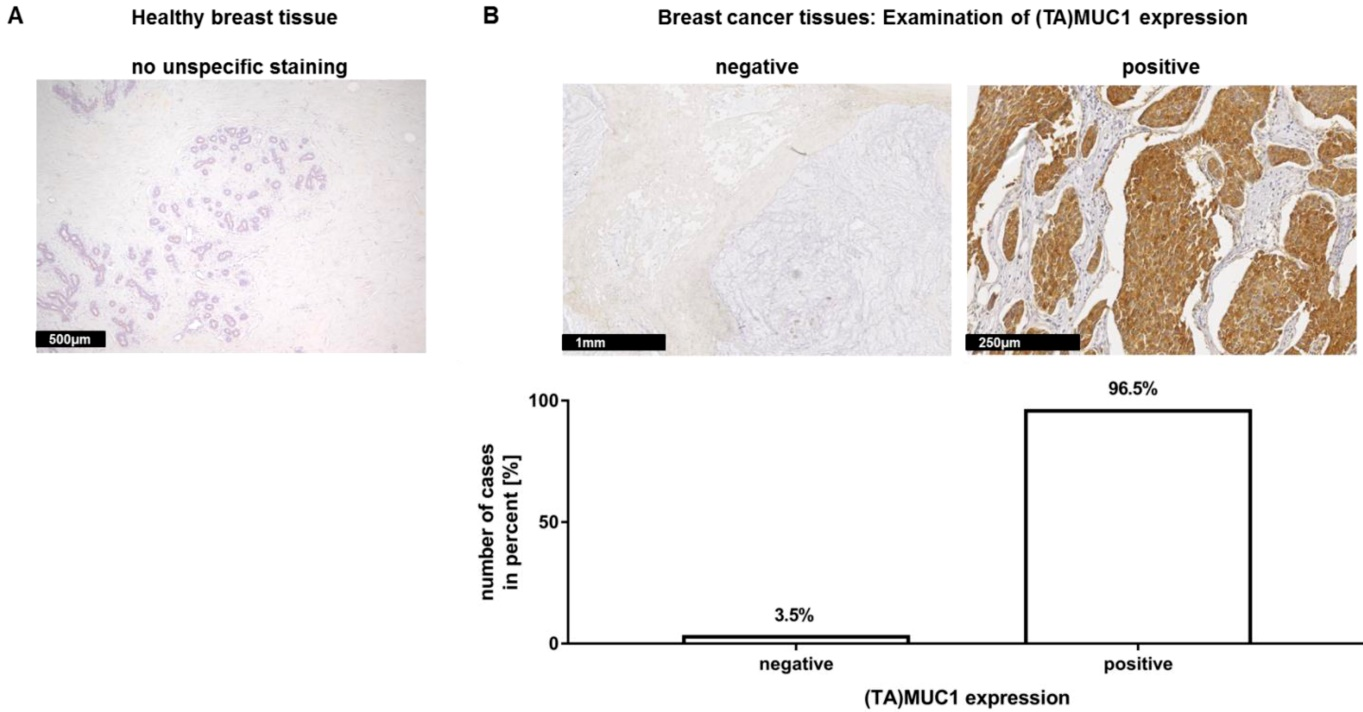
**Immunohistochemical staining of (TA)MUC1 with GGSK-1/30 in human breast cancer specimens**. A collective of breast cancer tissue sections from 144 patients was examined for (TA)MUC1 specific staining. Paraffin sections of healthy breast tissue (**A**) and paraffin sections of hormone receptor positive breast tumors (**B**) were examined. Representative examples from 144 breast cancer tissue sections and 10 healthy mammary tissue sections are shown. (TA)MUC1=Tumor-associated MUC1.

In a recent publication, we have already shown that our mAb GGSK-1/30 stained highly specific tumorous tissue from TNBC patients 18. In this study, we examined a very large group of human hormone receptor positive (HR positive) breast cancer biopsies with the mAb GGSK-1/30 in cooperation with the Department of Obstetrics and Women's Health of the University Medical Center in Mainz. HR positive breast cancer patients represent the largest group of patients with 75% 19. The immunohistochemistry (IHC) analyses demonstrated again the diagnostic use of mAb GGSK-1/30 for the detection of breast cancer tissue. Therefore, 10 sections of healthy human breast tissue (**Figure 1A**) and 144 sections of HR positive breast cancer tissue (**Figure 1B**) were stained with GGSK-1/30. The staining of healthy breast tissue with GGSK-1/30 was negative in all cases. By contrast 96.5% of all breast cancer tissue sections were clearly positively stained with GGSK-1/30, 3.5% were negative.

**Figure 2:**
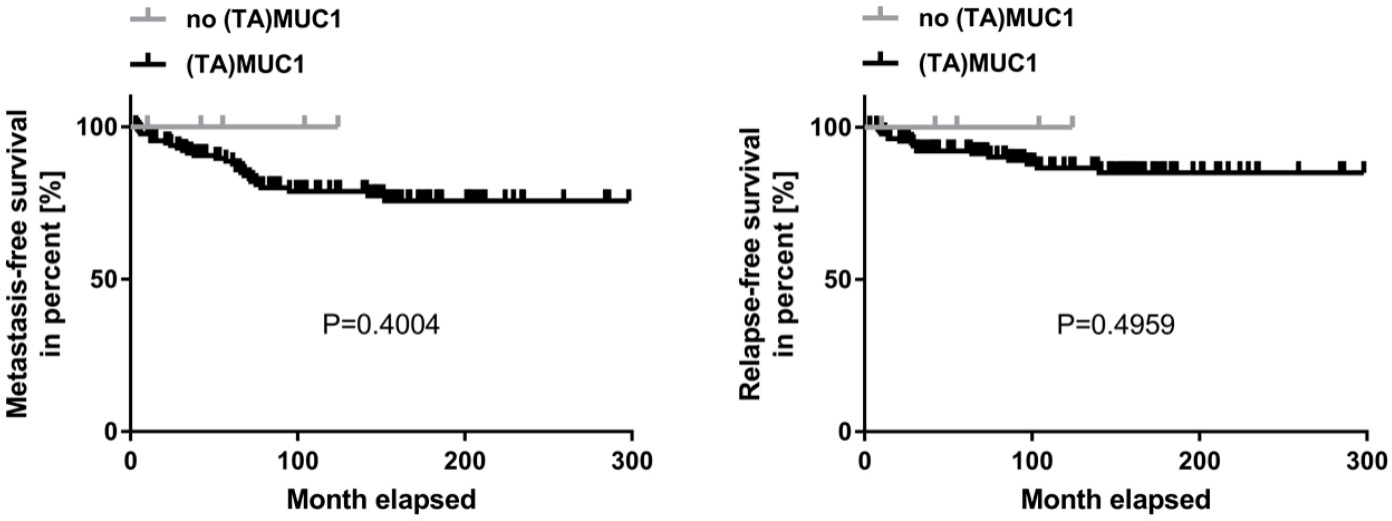
** Kaplan-Meier curve of metastasis-free and relapse-free survival comparing (TA)MUC1 expression and no (TA)MUC1 expressi**on. A collective of breast cancer tissue sections from 144 patients was examined for (TA)MUC1 specific staining. The status of (TA)MUC1 expression was correlated to metastasis-free or relapse-free survival with follow up patient data. Significance levels were calculated using Log-Rank-Test. (TA)MUC1=Tumor-associated MUC1.

A correlation analysis was carried out concerning (TA)MUC1 expression in relation to metastasis-free survival (MFS) and relapse-free survival (RFS) as well. The patient's cumulative MFS and RFS suggest that the (TA)MUC1 expression may be correlated with a comparatively poor prognosis (Figure 2). However, our data failed to show any prognostic significance of (TA)MUC1 expression neither in terms of MFS nor in terms of RFS in this cohort of HR positive breast cancer samples. This might be due to the small sample number in the subgroup of (TA)MUC1 negative expression. The immune histochemical data which confirmed an exclusive binding of mAb GGSK-1/30 to (TA)MUC1-glycopeptides indicated that this mAb can be used for the diagnosis of breast cancer. Whether (TA)MUC1 expression could be used as a prognostic marker, should be evaluated on the basis of a considerable larger collective.

**Figure 3:**
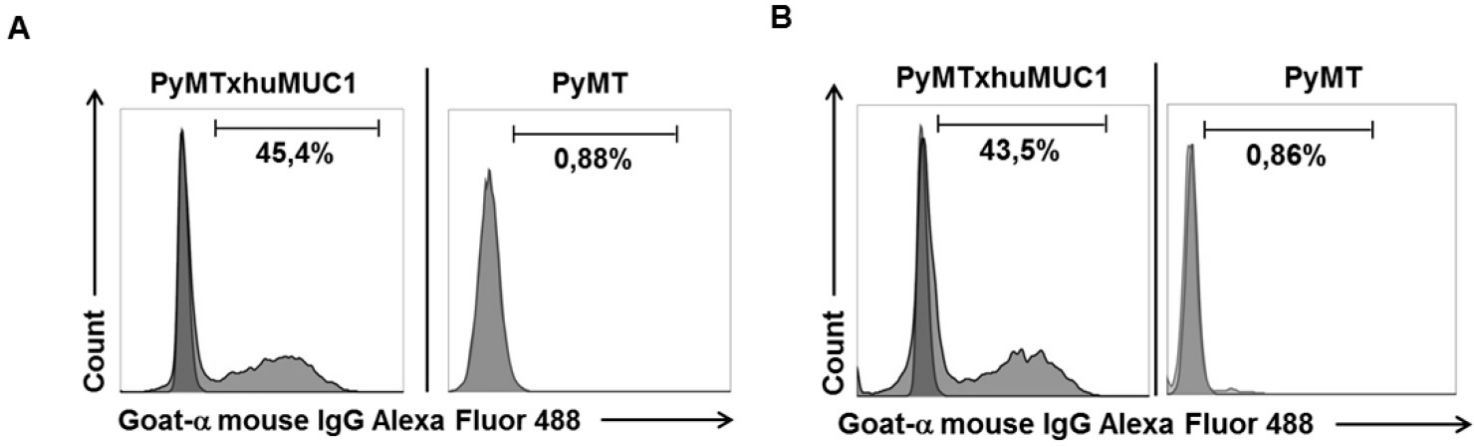
** Specific binding of mAb GGSK-1/30 and to huMUC1-expressing tumor cells*.*** Murine huMUC1-expressing PyMTxhuMUC1 tumor cells and PyMT tumor cells which did not express huMUC1 were incubated with A: GGSK-1/30 (1 µg/ml) and B: Df'-GGSK-1/30 (1 µg/ml). Binding was determined by FACS analysis. As control served the unspecific binding of the secondary antibody goat a-mouse IgG Alexa Fluor 488 to the tumor cells (dark grey).

After breast cancer diagnosis, positron emission tomography (PET) can be used to determine whether the cancer has spread to the lymph nodes or to other organs. During therapy, PET imaging can also be used in monitoring the effectiveness and response to the treatment(s). Women who have completed treatment, but remain at high risk for recurrence might also be good candidates for follow-up PET screening. The best-studied and most widely used clinical-grade PET tracer is 2-Fluor-2-desoxy-D-glucose (FDG) 20. However, false negative PET results due to low FDG uptake can easily occur in certain types of breast cancer, such as invasive lobular carcinoma. In addition, false positive results can occur during an inflammation. Therefore, the development of additional PET biomarkers is needed, which also aims to improve patient restaging information and to evaluate therapeutic efficacy 21. Thus, we analyzed whether the GGSK-1/30 mAb was applicable as a biomolecular imaging agent selectively binding to (TA)MUC1 in a preclinical breast cancer mouse model. We established a transplantable breast tumor model with murine breast cancer cells that express human MUC1 (PyMTxhuMUC1 cells) 18. These primary cancer cell lines were established from tumor biopsies of F1 PyMT (Tg(MMTVPyMT)634Mul 22) crossbred with human MUC1 (C57BL/6-TG(MUC1)79.24GEND/J 15) double transgenic mice (PyMTxhuMUC1 mice). Using FACS analysis we could show that the mAb GGSK-1/30 specifically binds to these human MUC1 expressing murine cancer cells (**Figure 3A**). As negative control served murine breast cancer cells that did not express human MUC1 after isolation from PyMT (Tg(MMTVPyMT)634Mul mice. For in vivo imaging studies GGSK-1/30 was radiolabeled with ^89^Zr. Long-lived PET nuclides like ^89^Zr are of great interest for ImmunoPET imaging and are ideal candidates for radiolabeling mAbs 23,24. ^89^Zr is advantageous because it remains in the cells after internalization of the mAb conjugate, resulting in improved tumor image contrast accumulation. In addition, its half-life of about 78 hours allows binding to the target over a longer period of time, which correlates well with the long biological half-life of mAbs 25. We used hydroxamate groups of desferrioxamine (Df') as chelating agent for ^89^Zr. Coupling of the Df' chelator resulted in a ratio of 4.2 chelator moieties per antibody. Binding of Df'-GGSK-1/30 mAb to PyMTxhuMUC1 tumor cell lines was analyzed by FACS analysis. **Figure 3B** demonstrates that binding of Df'-GGSK-1/30 to PyMTxhuMUC1 tumor cells was not impaired upon coupling of the Df'.

**Figure 4:**
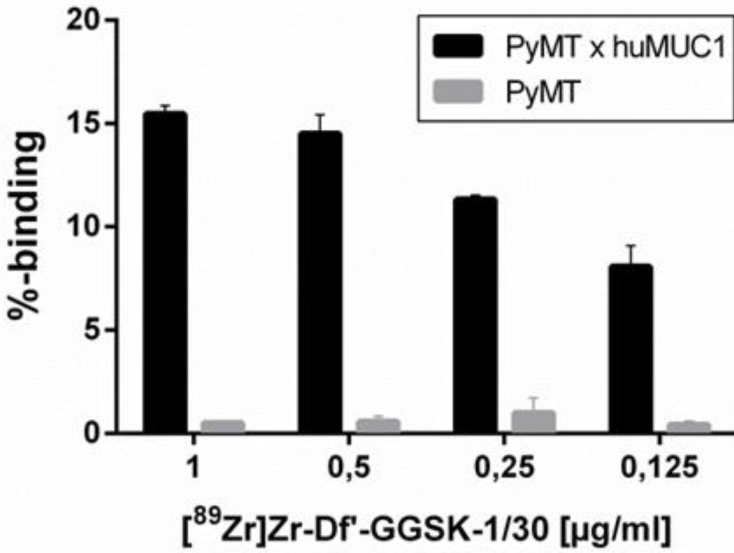
** Specific binding of [^89^Zr]Zr-Df'-GGSK-1/30 mAb to huMUC1-expressing tumor cells.** Murine PyMTxhuMUC1 tumor cells were incubated in the presence of decreasing concentrations of [^89^Zr]Zr-Df'-GGSK-1/30 (1-0.125 µg/ml) and binding was determined by FACS analysis. Murine PyMT tumor cell line which does not express huMUC1 served as negative controls.

The radiolabeling of Df'-GGSK-1/30 with ^89^Zr was performed at room temperature 26 with an overall yield of 73 % (**SI Figure 1**). After purification with a PD-10 desalting column, the radiochemical purity of [^89^Zr]Zr-Df'-GGSK-1/30 exceeded 95 % with an apparent specific activity of 6.1 GBq/µmol (**SI Figure 2**). [^89^Zr]Zr-Df'-GGSK-1/30 exhibited a high stability of >90 % after 3 days in human serum (HS) and 0.9 % NaCl solution (**SI Figure 3**). In 0.9 % NaCl solution [^89^Zr]Zr-DF'-GGSK-1/30 remained stable even after 3 days, while a slight decrease to 83 % intact conjugate after 7 days was observed in HS.* In vitro* binding of [^89^Zr]Zr-Df'-GGSK-1/30 to hu(TA)MUC1 was evaluated to verify the diagnostic potency of the radiolabeled conjugate in respect to first *in vivo* studies. A dose-dependent (>15 %) binding to murine PyMTxhuMUC1 breast tumor cells which express hu(TA)MUC1 could be observed, while [^89^Zr]Zr-Df'-GGSK-1/30 did not bind to the murine PyMT breast tumor cells (**Figure 4**).

**Figure 5:**
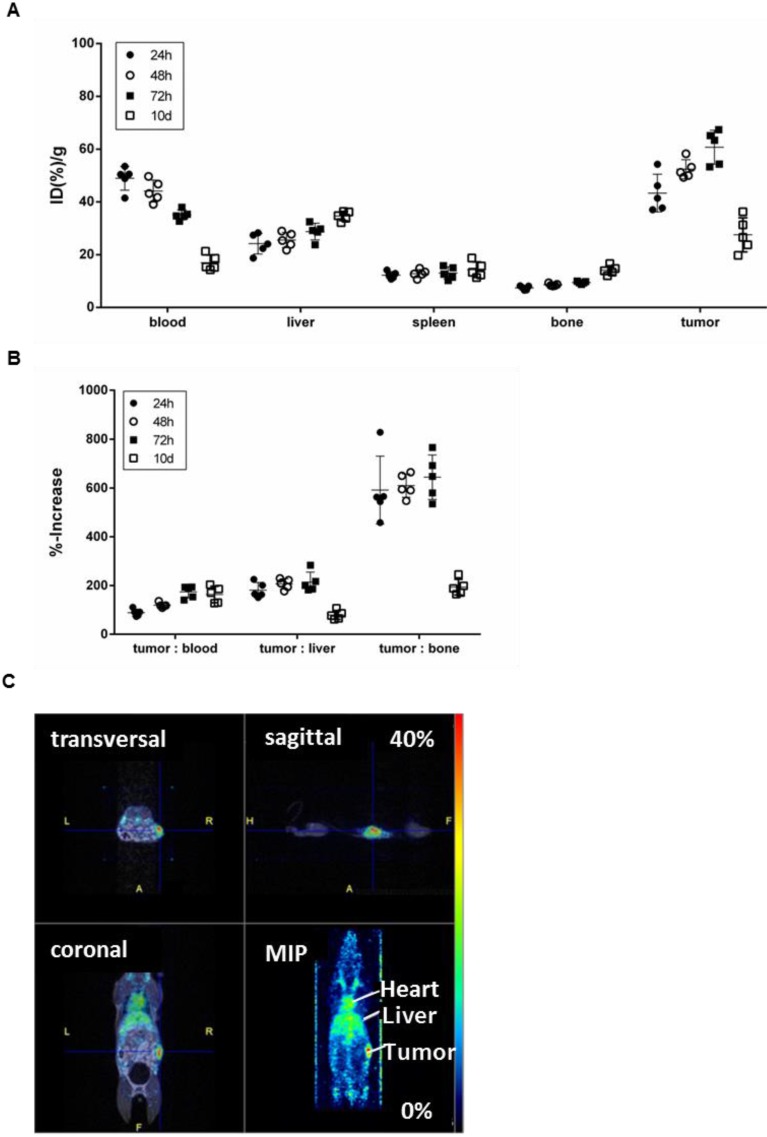
** Biodistribution of [^89^Zr]Zr-Df'-GGSK-1/30 in wild type mice bearing PyMTxhuMUC1 breast tumors.** C57BL/6N mice bearing a PyMTxhuMUC1 breast tumor transplant subcutaneously on the right flank were treated with [^89^Zr]Zr-Df'-GGSK-1/30 mAb (80 µg, 1 MBq) i.p. (n=20). After 24 h, 48 h, 72 h and 10 d the distribution of the radioconjugate (A) and the tumor/non-target-tissue ratios (B) were determined (ID(%)/g(tumor):ID(%)/g(blood, liver, bone)*100=%-increase). (C) PET images from a representative breast tumor-bearing mouse after 72 h. Abbreviations: tu.: tumor, he: heart, li.: liver; MIP: Maximum Intensity Projection.

To confirm the specificity of [^89^Zr]Zr-DF'-GGSK-1/30 for hu(TA)MUC1 *in vivo* and to assess its usage as a future diagnostic tool for huMUC1-expressing breast cancers, the radioconjugate was administered i.p. in C57BL/6N mice bearing PyMTxhuMUC1 breast tumor cells subcutaneously on the right flank. After 24 hours, 48 hours, 72 hours and 10 days analyses of the biodistribution were carried out. Additionally, PET imaging was performed after 72 hours (**Figure 5**). The highest amount of [^89^Zr]Zr-Df'-GGSK-1/30 was detected after 72 hours in the tumor (>55 %ID/g). Uptake values in other tissues (lung, heart, spleen, pancreas, stomach, intestines, kidneys, lymph nodes, mammary glands, muscle) were below 20 %ID/g(tissue) (**SI Figure 4**). The concentration of the [^89^Zr]Zr-Df'-GGSK-1/30 in blood decreased steadily while increasing amounts of [^89^Zr]Zr-Df'-GGSK-1/30 accumulated in the tumor. The radioconjugate showed predominant hepatobiliary excretion with increasing uptake values over time from 22 to 38 %ID/g (liver). The uptake values in bone tissues steadily increased (**Figure 5A**) due to the slight degradation of the Zr-Df'-complex *in vivo*, which is known for ^89^Zr-radiolabeled Df'-conjugated antibodies 26-28. An exact calculation of the tumor-to-tissue ratio revealed a comparatively high tumor accumulation (**Figure 5B**). In agreement with these findings, PET imaging after 72 hours demonstrated a strong accumulation of [^89^Zr]Zr-Df'-GGSK-1/30 in the tumor (**Figure 5C**).

**Figure 6:**
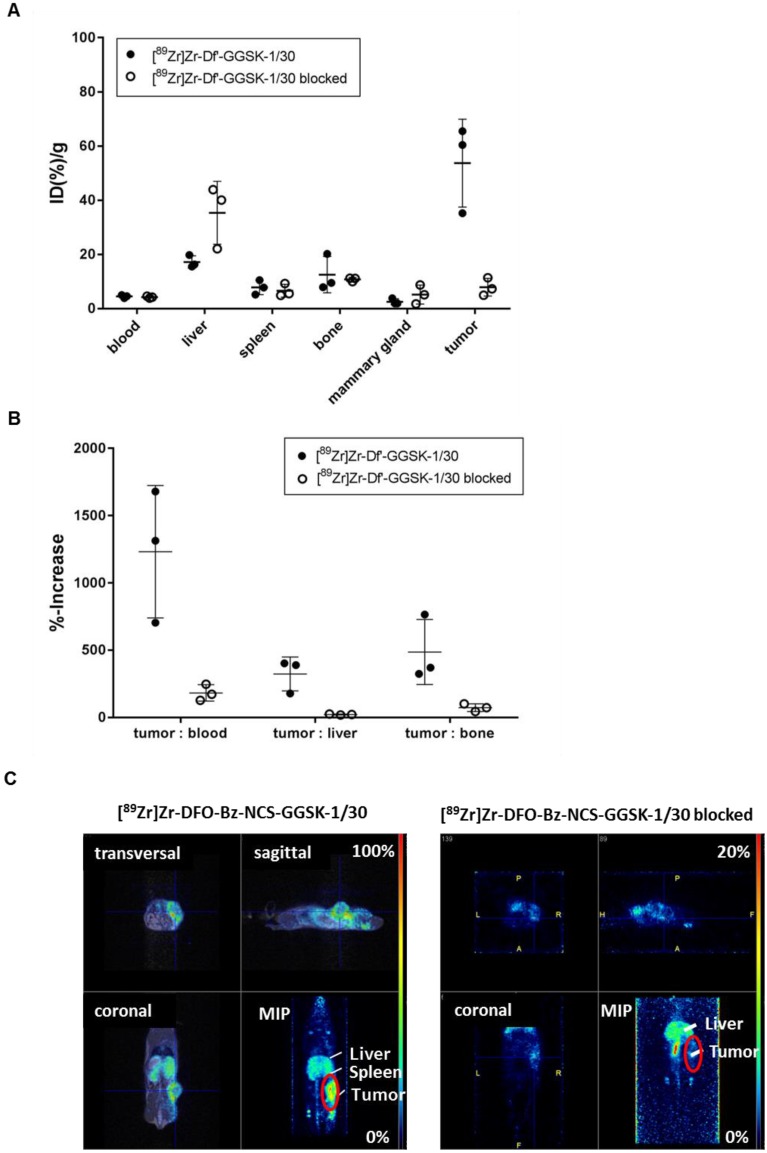
** Selective binding of [^89^Zr]Zr-Df'-GGSK-1/30 to hu(TA)MUC1 expressed by PyMTxhuMUC1 tumors.** HuMUC1-transgenic mice bearing a PyMTxhuMUC1 breast tumor transplant subcutaneously on the right flank were treated i.p. with [^89^Zr]Zr-Df'-GGSK-1/30 (80 µg, 2.5 MBq, black dots: ●), previously saturated with 1200 molar excess of the corresponding glycopeptide: [^89^Zr]Zr-Df'-GGSK-1/30 blocked (50 µg, 0,46 MBq, open circles: ○)**.** After 72 h the distribution of the radioconjugate was determined (A), the tumor/non-target-tissue ratios (B) were determined (ID(%)/g(tumor):ID(%)/g(blood, liver, bone)*100=%-increase) and PET imaging was performed with representative mice (B). Maximum Intensity Projections (MIPs) are shown. Abbr.: tu: tumor, ki: kidney, li: liver.

Potential diagnostic and therapeutic usage of [^89^Zr]Zr-Df'-GGSK-1/30 in breast cancer patients requires that unspecific binding to normally glycosylated huMUC1 on healthy tissue should be largely excluded. To investigate impact of unspecific binding on tumor accumulation, PyMTxhuMUC1 tumor cells were transplanted into huMUC1-transgenic mice carrying huMUC1 on all epithelial cells. Maximum accumulation of [^89^Zr]Zr-Df'-GGSK-1/30 in the tumor could be observed 72 hours after i.p. application in wild type mice. Therefore, analyses of *in vivo* biodistribution and PET imaging were performed in the huMUC1-transgenic mice at this time point. As additional control concerning the specificity of the mAb GGSK-1/30 for hu(TA)MUC1, mice were injected with [^89^Zr]Zr-Df'-GGSK-1/30 which had been blocked before with a 1200-fold molar excess of its specific hu(TA)MUC1-glycopeptide antigen 12. **Figure 6A** shows the uptake values of [^89^Zr]Zr-Df'-GGSK-1/30 (%ID/g (tissue)) for blood, spleen, liver, bones, tumor tissue and mammary glands. The low unspecific uptake values (less than 10 %ID/g) of the radiolabeled mAb in the mammary glands, which overexpress normal huMUC1 are similar to other non-target tissues, whereas up to 65 %ID/g could be observed in the tumor. An exact calculation of the tumor-to-tissue ratio revealed a comparatively high tumor accumulation (**Figure 6B**). These data demonstrate again that the unique mAb GGSK-1/30 binds to hu(TA)MUC1 containing the aberrant glycosylation pattern whereas normal huMUC1 expressed on healthy cells are not bound. Blocking of radiolabeled mAb by its specific hu(TA)MUC1-glycopeptide prevented binding to tumor tissue demonstrating again the antigen specificity of GGSK-1/30 for hu(TA)MUC1. A detailed presentation of the biodistribution of the [^89^Zr]Zr-Df'-GGSK-1/30 is shown in **SI Figure 5**. These analyses revealed an exceptional specificity of mAb GGSK-1/30 for hu(TA)MUC1 in vivo and were further supported *in vivo* by PET imaging (**Figure 6C**).

## Discussion

Early and specific detection of breast cancer remains a challenge in oncology. Intensive efforts are being made to identify the biological processes and new targets for TNBC. Molecular imaging of these targets may aid target identification, drug development, and in predicting and evaluating response to therapy 20. GGSK-1/30 is characterized by the fact that it exclusively recognizes a clearly defined synthetic MUC1-derived glycopeptide which was concomitantly demonstrated to block its binding to human breast cancer cells 29. In comparison SM3 and HMFG1 were induced against partial deglycosylated MUC1 from human milk. The binding epitope is the PDTRP amino acid sequence of the MUC1 tandem repeat. Due to the microheterogeneity of these antigens, the induced antibodies are not sufficiently specific to differentiate between physiological MUC1 and (TA) MUC1 30,31. The exceptional specificity of GGSK-1/30 for hu(TA)MUC1 combined with radiolabeling to the long lived isotope ^89^Zr allowed the generation of an innovative diagnostic tool characterized by high tumor accumulation to visualize hu(TA)MUC1 expression on breast cancer manifestations via PET imaging technology. In addition, GGSK-1/30 exhibited much higher and more specific tumor enrichment levels than previously reported for other anti-MUC1 mAbs 32,33. With these characteristics GGSK-1/30 represents a new promising tool concerning clinical studies for molecular imaging of breast cancer that might also be used for radiotherapeutic approaches since the mAb meets the key foundations for effective radioimmunotherapy: A high and tumor-specific accumulation of the radiopharmaceutical, as well as a low dose rate for the patients to avoid collateral damage from surrounding healthy tissue 34. Especially, recent studies have shown that (TA)MUC1 due to its strong expression in HR-positive 35,36, HER2/neu-positive breast tumors 14 and in TNBCs 11, is crucially involved in the development of resistance to the clinically used adjuvant therapies (tamoxifen, trastuzumab, systemic chemotherapy). The combinatory therapeutic use of anti-(TA)MUC1 antibody drugs with common therapeutic agents in adjuvant therapy could therefore increase their clinical effect.

## Conclusion

Hu(TA)MUC1 is a tumor-specific antigen on breast cancer cells with an exceptionally high diagnostic and potential prognostic value/importance. Immunizations against hu(TA)MUC1 enabled us to generate a unique antibody that specifically recognizes hu(TA)MUC1 glycopeptides on breast cancer cells. The specific immunohistochemical staining of breast cancer tissue with the mAb GGSK-1/30 confirmed that (TA)MUC1 represents a promising marker for diagnosis and most likely prognosis 37. Especially, due to its overexpression in 90% of all breast cancer patients 10,38 and in 94% of TNBC patients, 11 as well as the clear association of high expression with metastases and poor survival 39. The radiolabeled derivative [^89^Zr]Zr-Df'-GGSK-1/30 demonstrated high *in vivo* stability and highly selective and tumor-specific accumulation which resulted in high contrast PET imaging. Thus, GGSK-1/30 represents a promising PET-tracer for clinical studies on molecular imaging in early diagnosis and/or in therapy-accompanying control examinations of breast cancer patients undergoing systemic therapies. In conclusion, the mAb GGSK-1/30 represents a platform, which can be used (i) as a diagnostic tool for the detection of hu(TA)MUC1 in early breast cancer diagnosis, (ii) as a prognostic biomarker, (iii) as a companion diagnostic during therapy and (iv) in future perspective in radioimmunotherapy.

## Supplementary Material

Supplementary figures and tables.

## Figures and Tables

**Table 1:** Patientscharacteristics. Clinicopathological characteristics of hormone receptor positive patients who were treated at the Department of Obstetrics and Women's Health of University Medical Center Mainz (N=144). NST=invasive carcinoma of no special type, pT=primary tumor, N=number, (TA)MUC1=tumor-associated MUC1.

Characteristics	N	%
Tumor type		
NST	95	66
Invasive lobular	23	16
Invasive tubular	6	4.2
mucinous	2	1.4
other	18	12.4
pT stage		
pT1	54	37.5
pT2	74	51.4
pT3	2	1.4
pT4	14	9.7
Histological grade		
G I	15	10.4
G II	105	72.9
G III	24	16.6
Estrogen receptor status		
Negative	0	0
Positive	144	100
Progesterone receptor status		
Negative	17	11.9
Positive	127	88.1
Lymph node status		
Negative	40	27.8
Positive	104	72.2
(TA)MUC1		
(TA)MUC1 expression	139	96.5
no (TA)MUC1 expression	5	3.5

## Abbreviations

MUC1: mucin1
hu: human
TA: tumor-associated
ID: injected dose
mAb: monoclonal antibody
