# Supplementary material for: Evaluation of a novel monoclonal antibody against tumor-associated MUC1 for diagnosis and prognosis of breast cancer
Source: Int J Med Sci. 2019 Aug 14;16(9):1188–98. doi: 10.7150/ijms.35452 (PMC6775261; doi:10.7150/ijms.35452)
Supplement: Supplementary file 1 — Supplementary figures and tables. [file ijmsv16p1188s1.pdf]

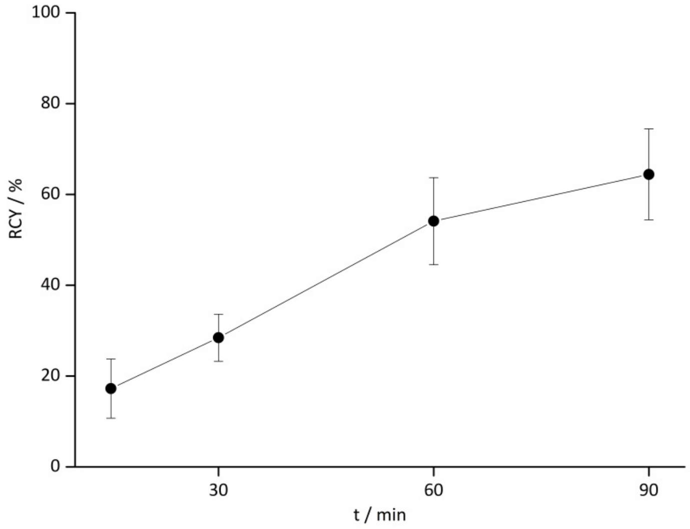

SI Figure 1: Radiolabeling kinetics with ^{89}Zr of Df'-GGSK-1/30 in 0.5 M HEPES (pH 7). Df'-GGSK-1/30 was radiolabeled with ^{89}Zr in HEPES buffer (0.5 M, pH = 7) at room temperature in a volume of 2.5-3 ml with gentle agitation for 90 min. The radiochemical yield (RCY) was checked by radio-thin-layer chromatography (Radio-TLC) and analyzed by Raytest radio detector GABI STAR. The radiolabelled compound was purified by a PD-10 column using a 0.9% NaCl solution as eluent.

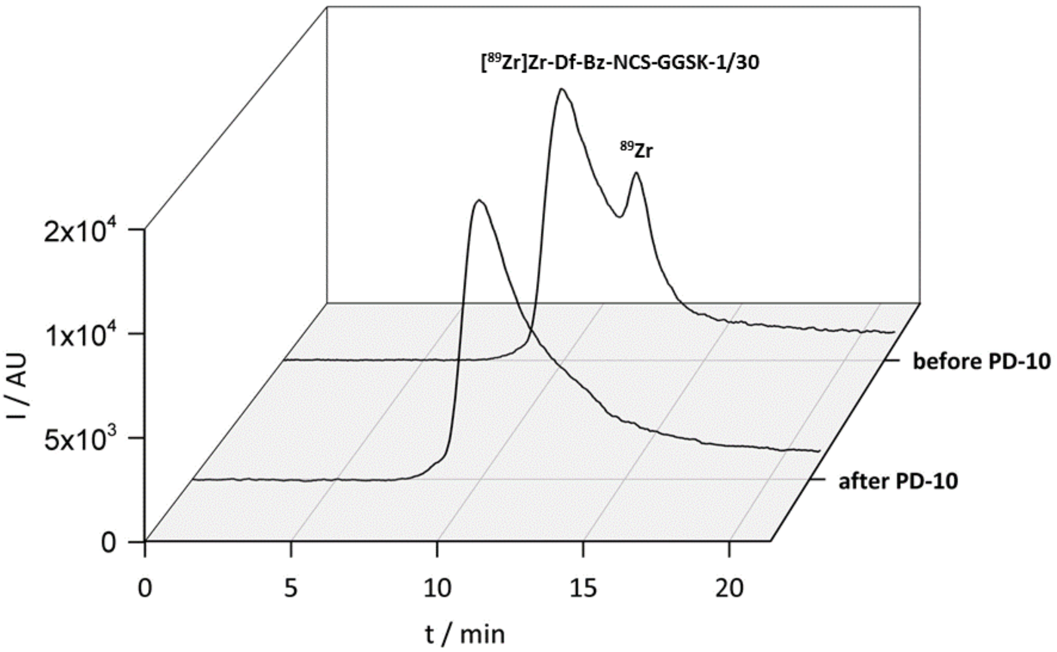

SI Figure 2: HPLC diagram of [^{89}Zr]Zr-Df'-GGSK-1/30 using BioSep SEC-S 2000 column (Phenomenex®) with 0.05 M sodium phosphate (pH 7) as mobile phase (1 ml/min) before and after purification using size exclusion chromatography on PD-10 column.

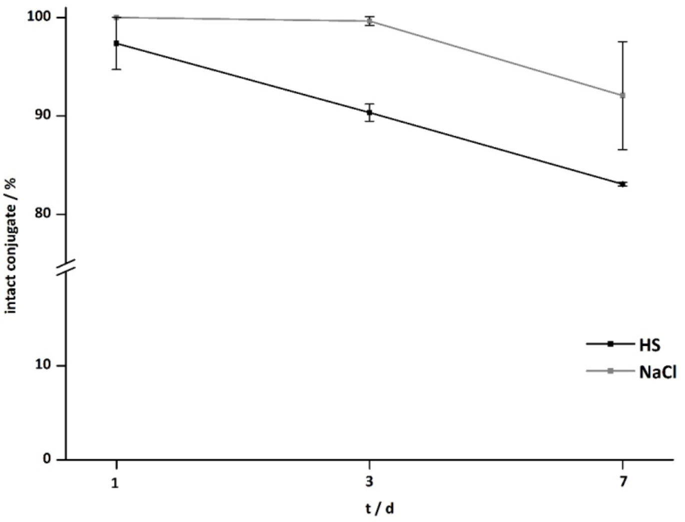

SI Figure 3: Stability of [^{89}Zr]Zr-Df'-GGSK-1/30 in human serum (black line) and sodium chloride solution. In vitro stability studies of [^{89}Zr] Zr-Df'-GGSK-1/30 were performed in human serum (HS, Sigma-Aldrich®, from human male AB plasma) and sodium chloride (NaCl, 0.9%) (n = 3) carried out. The samples were incubated at 37 ° C and samples of 2 µl were analyzed at various times (1 d, 3 d, 7 d) by radio-DC using citrate buffer. R (0.01M, pH = 4) and analyzed with the radio detector GABI STAR by Raytest. HPLC monitoring was performed on a Merck (LaChrom) pump (Hitachi L7100 pump; UV detector: L7400) using a BioSep SEC-S 2000 column (Phenomenex®) with 0.05 M sodium phosphate (pH = 7) as a mobile phase. (1 ml / min).

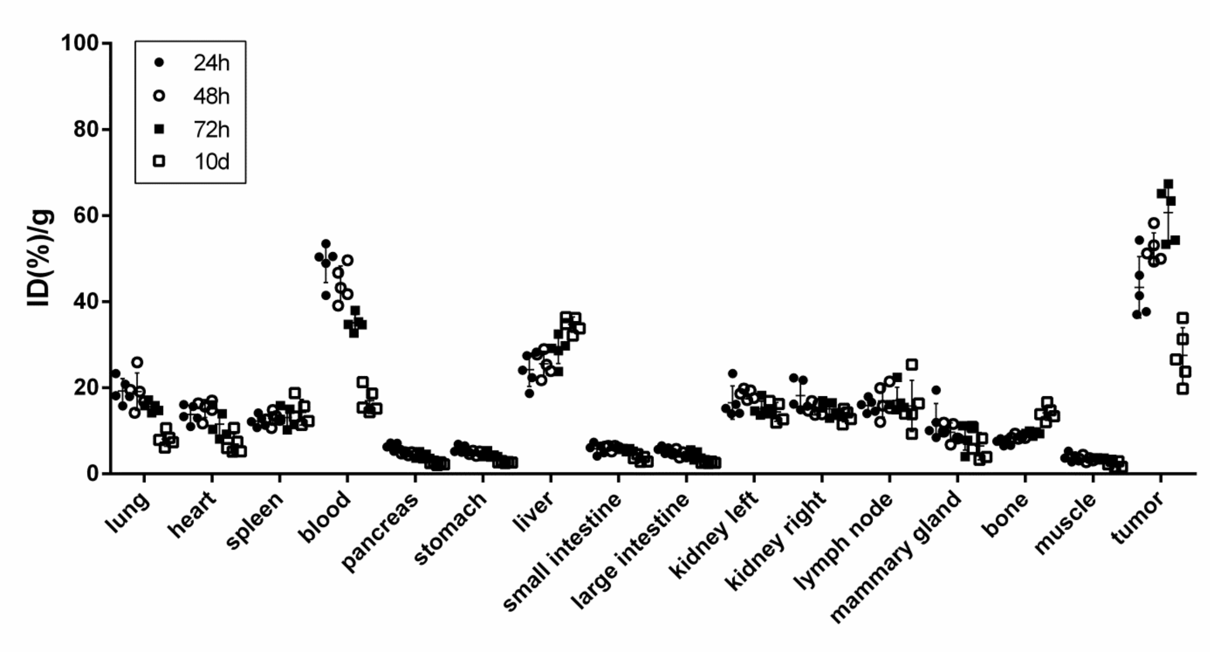

SI Figure 4: *Ex vivo* biodistribution of radiolabeled GGSK-1/30 mAb in C57BL/6N mice bearing PyMTxhuMUC1 tumors after 24 h, 48 h, 72 h and 10 d. C57BL/6N mice bearing a PyMTxhuMUC1 breast tumor transplant s.c. on the right flank were treated with [^{89}Zr]Zr-Df'-GGSK-1/30 mAb (80 µg, 1 MBq) i.p. (n=5). After 24 h, 48 h, 72 h and 10 d the distribution of the radio-labelled mAb was determined.

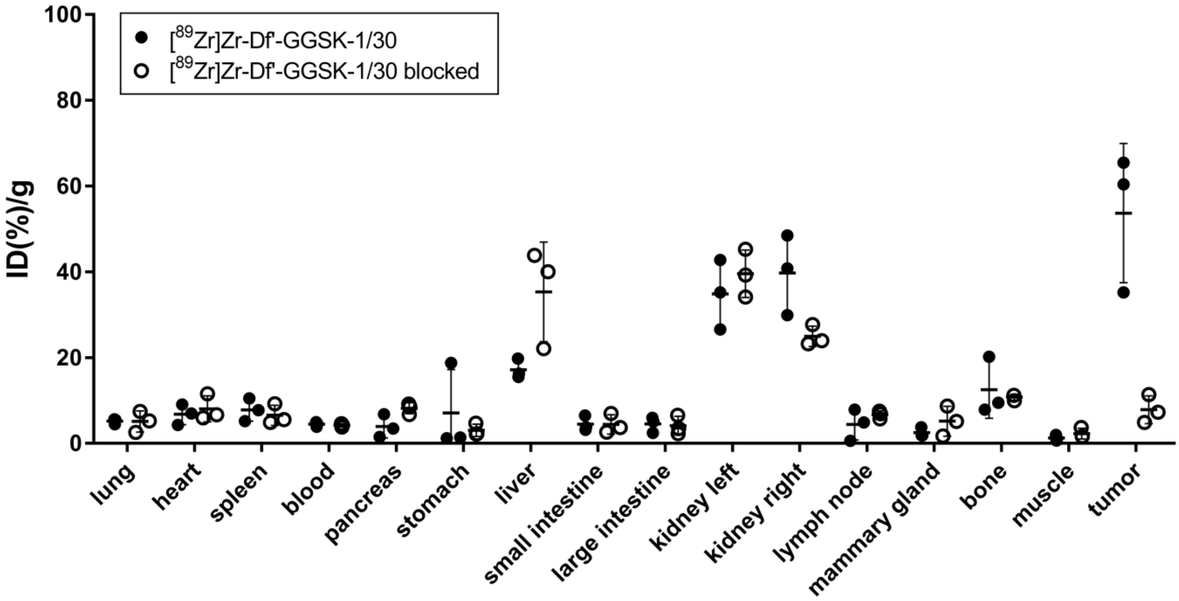

SI Figure 5: *Ex vivo* biodistribution of radiolabeled GGSK-1/30 mAb, neutralized and unspecific IgG1 antibody in huMUC1-transgenic mice bearing PyMTxhuMUC1 tumors after 72 h. HuMUC1-transgenic mice bearing a PyMTxhuMUC1 breast tumor transplant s.c. on the right flank were treated i.p. with [^{89}Zr]Zr-Df'-GGSK-1/30 mAb (80 µg, 2.5 MBq), blocked [^{89}Zr]Zr-Df'-GGSK-1/30 (50 µg, 0,46 MBq)**,** or [^{89}Zr]Zr-Df'-IgG1 isotype control (80 µg, 2.3 MBq)**.** After 72 h the distribution of the radio-labelled mAb was determined.
